# Inhibition of the Lipid Droplet–Peroxisome Proliferator-Activated Receptor α Axis Suppresses Cancer Stem Cell Properties

**DOI:** 10.3390/genes12010099

**Published:** 2021-01-14

**Authors:** Kenta Kuramoto, Masahiro Yamamoto, Shuhei Suzuki, Keita Togashi, Tomomi Sanomachi, Chifumi Kitanaka, Masashi Okada

**Affiliations:** 1 Department of Molecular Cancer Science, Yamagata University School of Medicine, Yamagata 990-9585, Japan; kenta.kuramoto@gmail.com (K.K.); masahiro@med.id.yamagata-u.ac.jp (M.Y.); snowdrop_ruby@yahoo.co.jp (S.S.); katyl0l6@yahoo.co.jp (K.T.); t-sanomachi@med.id.yamagata-u.ac.jp (T.S.); ckitanak@med.id.yamagata-u.ac.jp (C.K.); 2 Department of Clinical Oncology, Yamagata University School of Medicine, Yamagata 990-9585, Japan; 3 Department of Ophthalmology and Visual Science, Yamagata University School of Medicine, Yamagata 990-9585, Japan; 4 Research Institute for Promotion of Medical Sciences, Yamagata University Faculty of Medicine, Yamagata 990-9585, Japan

**Keywords:** peroxisome proliferator-activated receptor, cancer-initiating cell, lipid droplet, pancreatic cancer, colorectal cancer, re-esterification, lipolysis

## Abstract

Cancer stem cells (CSCs), having both self-renewal and tumorigenic capacity, utilize an energy metabolism system different from that of non-CSCs. Lipid droplets (LDs) are organelles that store neutral lipids, including triacylglycerol. Previous studies demonstrated that LDs are formed and store lipids as an energy source in some CSCs. LDs play central roles not only in lipid storage, but also as a source of endogenous lipid ligands, which are involved in numerous signaling pathways, including the peroxisome proliferator-activated receptor (PPAR) signaling pathway. However, it remains unclear whether LD-derived signal transduction is involved in the maintenance of the properties of CSCs. We investigated the roles of LDs in cancer stemness using pancreatic and colorectal CSCs and isogenic non-CSCs. PPARα was activated in CSCs in which LDs accumulated, but not in non-CSCs, and pharmacological and genetic inhibition of PPARα suppressed cancer stemness. In addition, inhibition of both re-esterification and lipolysis pathways suppressed cancer stemness. Our study suggested that LD metabolic turnover accompanying PPARα activation is a promising anti-CSC therapeutic target.

## 1. Introduction

Cancer stem cells (CSCs) comprise a small population of cells with self-renewal and tumorigenic capacity, functioning in the tumorigenesis, recurrence, and heterogeneity of tumors. CSCs utilize an energy metabolism system different from that used by non-CSCs, which compose most of the tumor. Non-CSCs preferentially employ glycolysis rather than mitochondrial oxidative phosphorylation (OXPHOS) to produce energy even in a normoxic state. On the other hand, energy is produced by both glycolysis and mitochondrial respiration in many CSCs [1,2,3,4,5]. Based on these reports, the OXPHOS of CSCs has attracted attention as a therapeutic target [3,5,6,7,8,9]. ATP production utilizing OXPHOS requires a large amount of acetyl-CoA, and acetyl-CoA is supplied not only by synthesis from the end product of glycolysis, pyruvic acid, but also by the β-oxidation of fatty acids. Fatty acids are exogenously (from nutrients such as meals) or endogenously (novel synthesis and lipolysis) supplied and used for β-oxidation. In some cancers, greater incorporation of fatty acids, lipid biosynthesis, cholesterol synthesis, and β-oxidation, as well as an increase in the number of lipid droplets (LDs), are observed [10,11,12].

LDs were previously considered to be organelles which accumulate lipids, mainly cholesterol esters and triacylglycerols (TAGs) [13], but recently they have been recognized as organelles with independent functions as regulators of lipid metabolism and numerous signaling pathways [14,15,16,17]. There are few studies on the association between the maintenance of cancer stemness and lipid accumulation in LDs, but there is growing evidence that the number of LDs in CSCs—including those in colorectal cancer, ovarian cancer, and glioblastoma—is higher than that in non-CSCs, and that lipid molecules are important for CSC tumorigenicity [10,18,19,20,21,22]. In addition, previous studies reported that LDs play an important role as an energy source in some CSCs [19,23]. However, it is unclear whether LD-derived ligands are involved in the maintenance of cancer stemness. Lipolysis products of LDs activate many signaling pathways and transcription through peroxisome proliferator-activated receptors (PPARs), which are nuclear receptors. PPARα, a member of the PPAR family, is activated by fatty acids, resulting in the promotion of mitochondrial biosynthesis [24]. However, little is known about the association between the maintenance of cancer stemness and the LD–PPARα axis. In this study, using pancreatic and colorectal CSCs, we investigated whether the LD–PPARα axis is involved in the maintenance of CSC properties.

## 2. Materials and Methods

### 2.1. Reagents and Antibodies

GW6471 and Atglistatin were purchased from Cayman Chemical (Ann Arbor, MI, USA). GSK3787 was purchased from Abcam (Cambridge, UK). GW9662 was purchased from FUJIFILM Wako Chemicals (Osaka, Japan). A922500 was purchased from Sigma-Aldrich (St. Louis, MO, USA). Antibodies against carnitine palmitoyltransferase 2 (CPT2), electron transfer flavoprotein subunit α (ETFα), electron transfer flavoprotein dehydrogenase (ETFDH), Acox1, and VLCAD were kindly gifted by T. Osumi (University of Hyogo) [25,26,27]. ADRP (PRIN2; sc-377429) and adipose triglyceride lipase (ATGL) (sc-365278) were purchased from Santa Cruz Biotechnology (Dallas, TX, USA). Antibodies against SOX2 (#3579), Nanog (#4903), Oct4 (#2890), and glyceraldehyde-3-phosphate dehydrogenase (GAPDH; #5174) were purchased from Cell Signaling Technology (Danvers, MA, USA). Horseradish peroxidase (HRP)-conjugated anti-rabbit IgG and anti-mouse IgG secondary antibodies were purchased from Jackson ImmunoResearch (West Grove, PA, USA).

### 2.2. Cell Culture

The cancer stem cells (CSCs) used in this study (PANC-1, PSN-1, SW620, HT29, WiDr, and SW480) were maintained as monolayer stem cell cultures [28,29]. Briefly, the cells were cultured on collagen-I-coated dishes (IWAKI, Tokyo, Japan) in stem cell culture medium (DMEM/F-12 supplemented with 1% B27 (Thermo Fisher Scientific, Waltham, MA, USA), 20 ng/mL of EGF and FGF2 (Peprotech, Rocky Hill, NJ, USA), D-(+)-glucose (final concentration, 26.2 mM), L-glutamine (final concentration, 4.5 mM), 100 units/mL of penicillin, and 100 mg/mL of streptomycin). The stem cell culture medium was changed every 3 days, and EGF and FGF2 were added to the stem cell culture medium daily. To obtain isogenic non-CSC counterparts, the CSCs were induced to lose their stemness by culturing in DMEM/F-12 medium supplemented with 10% fetal bovine serum (Thermo Fisher Scientific), 100 units/mL of penicillin, and 100 mg/mL of streptomycin for 1 week. Then, the cells were used in the experiments in this study as non-CSCs.

### 2.3. Gene Silencing by siRNA

siRNA against human *PPARA* (#1: HSS108289, #2: HSS108290, #3: HSS108291) and Medium GC Duplex #2 of Stealth RNAi™ siRNA Negative Control Duplexes (non-targeting control) were obtained from Thermo Fisher Scientific (Waltham, MA, USA). Cells were transiently transfected with siRNA using Lipofectamine RNAiMAX™ (Thermo Fisher Scientific) according to the manufacturer’s instructions.

### 2.4. Immunoblot Analysis

Immunoblot analysis was performed as previously described [7,30]. Briefly, cells were harvested and washed with ice-cold PBS. After centrifugation, the cell pellets were lysed in RIPA buffer (10 mM Tris/HCl (pH 7.4), 0.1% sodium dodecyl sulfate (SDS)), 1% Nonidet P-40, 0.1% sodium deoxycholate, 150 mM NaCl, 1 mM EDTA, 1.5 mM sodium orthovanadate, 10 mM sodium pyrophosphate, 10 mM sodium fluoride, and protease inhibitor cocktail set III (Sigma–Aldrich)), followed by the immediate addition of the same volume of 2× Laemmli buffer (125 mM Tris/HCl (pH 6.8), 4% SDS, 10% glycerol, and 10% 2-mercaptoethanol) and boiling at 95 °C for 10 min. Protein concentrations were measured using a BCA Protein Assay Kit (Thermo Fisher Scientific). Samples containing equivalent amounts of protein were separated by SDS-polyacrylamide gel electrophoresis and transferred to polyvinylidene difluoride membranes. The membranes were probed with the indicated primary antibodies and appropriate HRP-conjugated secondary antibodies, as recommended by the manufacturer of each antibody. To reprobe immunoblots, primary and secondary antibodies were stripped from the probed membrane using stripping buffer (2% SDS, 100 mM β-mercaptoethanol, 62.5 mM Tris-HCl (pH6.8)). After stripping, the membranes were washed with TBS-T and blocked with skim milk. Then, the membranes were reprobed with the appropriate antibodies. Immunoreactive bands were visualized using Immobilon Western Chemiluminescent HRP Substrate (Merck Millipore, Billerica, MA, USA) and detected by a ChemiDoc Touch device (Bio-Rad, Hercules, CA, USA).

### 2.5. LD Staining

Two days before the experiment, cells were plated on Geltrex-coated coverslips. To stain the LDs of WiDr cells, the cells were incubated with BODIPY FL C_12_ (Thermo Fisher Scientific) for 8 h. After washing the cells with PBS, they were fixed in 4% paraformaldehyde. For staining the LDs of PANC-1 cells, the cells were fixed with 4% paraformaldehyde and then stained with HCS LipidTOX Deep Red neutral lipid stain (Thermo Fisher Scientific) according to the manufacturer’s protocol. Cells were mounted using glycerol/PBS solution. Fluorescence images were acquired using a FLUOVIEW FV10i confocal laser-scanning microscope system (Olympus, Tokyo, Japan).

### 2.6. Reverse Transcription-PCR (RT-PCR)

Total RNA was extracted from cells using Trizol (Thermo Fisher Scientific), and 2 µg of total RNA was reverse transcribed using the PrimeScript RT Reagent Kit (Takara Bio Inc., Shiga, Japan) according to the manufacturer’s protocol. Target genes were amplified with Quick Taq HS DyeMix (Toyobo CO., Ltd., Osaka, Japan) using the following gene-specific primers: *GAPDH* forward 5′-ACCATCTTCCAGGAGCGAGAT-3′, *GAPDH* reverse 5′-TGACGAACATGGGGGCATC-3′, *PPARA* forward 5′-GGACAAGGCCTCAGGCTATC-3′, and *PPARA* reverse 5′-AACGAATCGCGTTGTGTGAC-3′. Quantification of the bands in the gels was performed by densitometry using Image J software (http://imagej.nih.gov/ij/).

### 2.7. Sphere Formation Analysis

The sphere formation assay was performed as previously described [31]. For primary sphere formation assays, cells treated with drugs in 35-mm collagen-I-coated dishes were washed with PBS to remove drugs completely. The cells dissociated into single cells by pipetting were serially diluted in the stem cell culture medium and seeded onto non-coated 96-well plates such that each well contained a single cell. The wells containing a single cell were marked under a microscope on the day after seeding, and cells were incubated for 6 more days to form tumorspheres. For secondary sphere formation analyses, primary spheres formed by cells seeded onto non-coated 12-well plates at a density of 5 × 10^2^ cells/well were collected 6 days after seeding. After dissociation of primary spheres by pipetting, single cells were seeded on non-coated 96-well plates the same as for primary sphere formation analysis. Wells containing a single viable cell were marked under a phase-contrast microscope on the next day, and 7 days after seeding the 96-well plate, the percentage of marked wells with a sphere relative to the total number of marked wells was calculated.

### 2.8. TAG Measurement Analysis

TAG measurement analysis was performed as previously described [27]. Briefly, after washing the cells with PBS, all lipids were extracted by the Folch method. Extracted lipids were resuspended in isopropanol. TAG levels were measured using a triglyceride E test Wako kit (FUJIFILM Wako Pure Chemical Corporation, Osaka, Japan) according to the manufacturer’s protocol. The intracellular TAG level was normalized by the total protein level.

### 2.9. Statistical Analysis

All data were expressed as means ± standard deviations. Differences were compared using a two-tailed Student’s *t*-test. For comparisons of more than two groups, data were analyzed using a one-way analysis of variance followed by Dunett’s test. *p*-Values < 0.05 were considered significant and are indicated with asterisks (*) in the figures.

## 3. Results

### 3.1. LDs Develop More in CSCs Than in Non-CSCs

**Figure 1:**
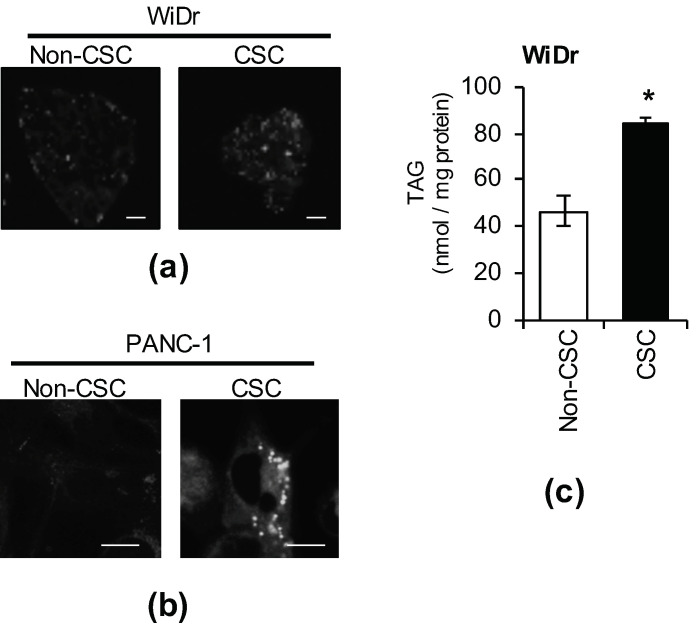
Lipid droplets (LDs) accumulate in cancer stem cells (CSCs). CSCs and non-CSCs were stained using BODIPY ((**a**), WiDr) or LipidTOX ((**b**), PANC-1). Photographs of the representative images are shown. Scale bars: 10 μm. (**c**) The intracellular triacylglycerol (TAG) quantities of CSCs and non-CSCs was measured. The values are means ± SDs from triplicate samples of a representative experiment. Similar results were obtained from three independent experiments. Similar results were obtained from two independent biological replicates. * *p* < 0.05 by the Student’s *t*-test.

Among CSCs, which play an important role in tumorigenicity, LD accumulation in those of colorectal cancer, ovarian cancer, and glioblastoma was reported to be higher than in non-CSCs [18,19,20]. Thus, we stained LDs with BODIPY FL C_12_ or LipidTOX neutral lipid dye in colorectal cancer stem cells (WiDr CSCs), pancreatic cancer stem cells (PANC-1 CSCs), and isogenic non-CSCs. LD development was observed more in CSCs than in non-CSCs (Figure 1a,b). In addition, the amount of TAGs, the main component of LDs, was higher in CSCs than in non-CSCs (Figure 1c). Based on the above, the number of LDs and intracellular TAG levels in CSCs were higher than those in isogenic non-CSCs.

### 3.2. The Peroxisome Proliferator-Activated Receptor α (PPARα) Pathway is Activated in CSCs

**Figure 2:**
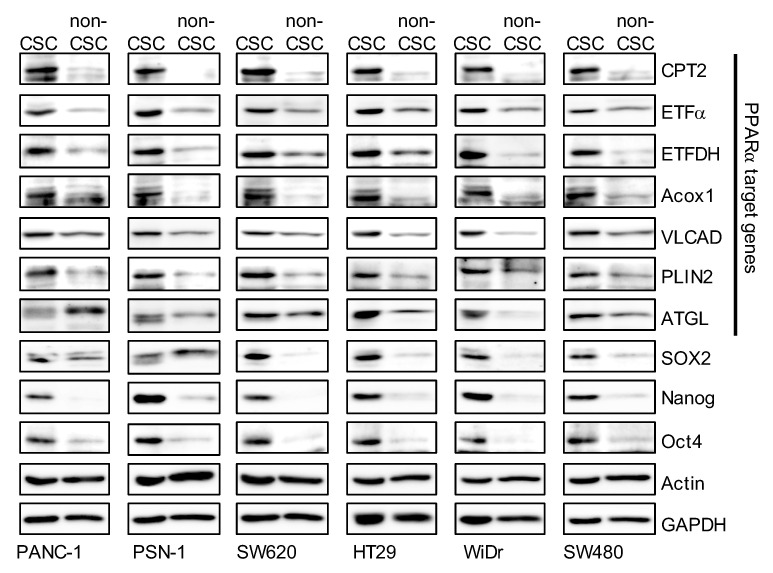
The expression levels of peroxisome proliferator-activated receptor α (PPARα) target genes in cancer stem cells (CSCs) were higher than in non-cancer stem cells (non-CSCs). Pancreatic-cancer-derived (PANC-1, PSN-1) and colorectal-cancer-derived (SW620, HT29, WiDr, and SW480) CSCs and non-CSCs were subjected to immunoblot analyses of the indicated proteins. Similar results were obtained from two independent biological replicates.

It is well known that LDs are organelles that store excess fatty acids as neutral lipids such as TAGs, and supply lipids as an energy substrate. Growing evidence over the past decade suggests that LDs function as a source of lipid ligands, which regulate many signaling pathways. As intracellular TAG levels in CSCs were higher than in non-CSCs, we hypothesized that the expression levels of lipid-metabolism-related proteins in CSCs are higher than in non-CSCs. To test this hypothesis, we compared the expression levels of lipid-metabolism-related proteins between CSCs and non-CSCs. The expression of carnitine palmitoyltransferase 2 (CPT2), electron transfer flavoprotein subunit α (ETFα), electron transfer flavoprotein dehydrogenase (ETFDH), acyl-CoA oxidase 1 (Acox1), very-long-chain specific acyl-CoA dehydrogenase, mitochondrial (VLCAD), perilipin 2 (PLIN2), and adipose triglyceride lipase (ATGL) were higher in CSCs than in non-CSCs (Figure 2). Of note, the expression of these genes is regulated by PPARα [32]. This suggests that the PPARα is activated more in CSCs than in non-CSCs.

### 3.3. Inhibition of the PPARα Suppresses the Expression of CSC Markers

**Figure 3:**
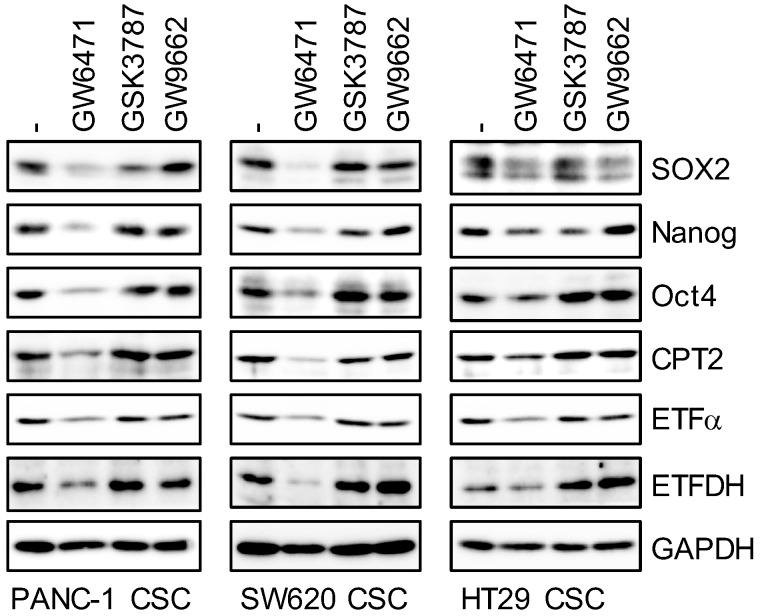
Pharmacological inhibition of PPARα by its antagonist GW6471 causes the loss of stem cell marker expression. Cells treated with 5 μM PPAR antagonist (GW6471: PPARα, GSK3787: PPARδ, and GW9662: PPARγ) were subjected to immunoblot analyses of indicated proteins. Similar results were obtained from two independent biological replicates.

Higher intracellular TAG levels and PPARα-regulated gene expression in CSCs led to the hypothesis that the LD–PPARα axis plays an important role in maintaining cancer stemness. To test this hypothesis, we examined whether activation of the PPAR is necessary for the maintenance of cancer stemness by selectively inhibiting the PPAR using several PPAR-specific antagonists. GW6471 was used to inhibit the PPARα, and PPARβ/δ and PPARγ antagonists (GSK3787: PPARβ/δ antagonist, GW9662: PPARγ antagonist) were used as controls. Inhibition of the PPARα suppressed the expression of typical transcription products of PPARα such as CPT2, ETFα, and ETFDH, whereas PPARδ and PPARγ antagonists did not inhibit the expression of these gene products (Figure 3). In addition, PPARα antagonists inhibited the expression of several stem cell markers (SOX2, Nanog, and Oct4), but PPARδ and PPARγ antagonists produced no specific pattern (Figure 3). This suggests that inhibition of the PPARα, which is activated by fatty acids, a TAG metabolite abundant in LDs, reduces the expression of stem cell markers in CSCs.

### 3.4. PPARα Suppression Inhibits the Sphere-Formation Ability of CSCs

**Figure 4:**
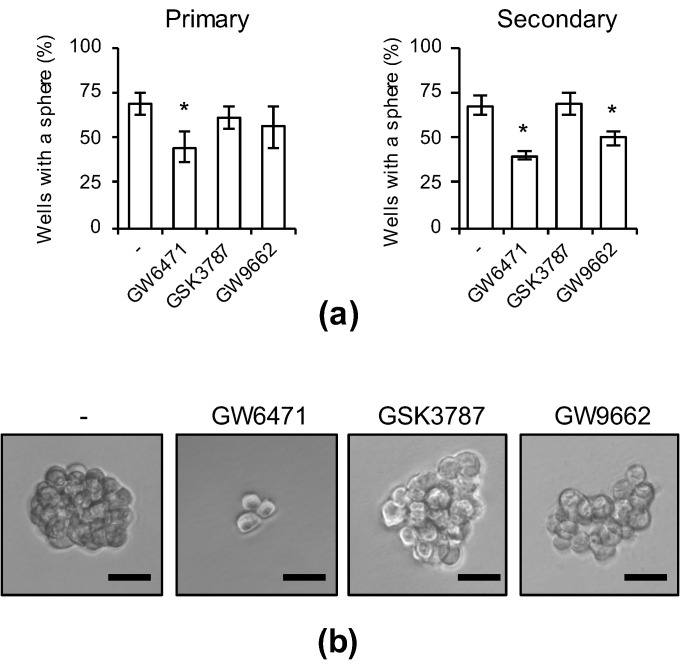
The effects of pharmacological PPARα inhibition on the sphere-forming ability. PANC-1 CSCs were treated with 5 μM PPAR antagonists for 10 days. After washing out the drugs, cells were analyzed in the serial sphere-formation assay. (**a**) The graphs show the means ± SDs from three independent experiments. (**b**) Representative photographs of primary spheres are shown. Similar results were obtained from three independent biological replicates. Bar: 50 μm. * *p* < 0.05 vs. control-treated cells by Dunnett’s test.

As the inhibition of PPARα activity strongly suppressed the expression of CSC markers, we next investigated whether the suppression of PPARα inhibits sphere-formation ability—an index of CSC properties. After treatment of cells with each PPAR antagonist, the drugs were removed and sphere formation was analyzed. The number of CSCs forming spheres significantly decreased in cells treated with the PPARα antagonist, but no decrease was noted in cells treated with PPARδ or PPARγ antagonists (Figure 4a,b). Continuous sphere-formation analysis in which secondary spheres were formed after dispersion of primary spheres using an enzyme was also performed, and the sphere-formation ability of cells treated with the PPARα antagonist was lower than that in the other antagonist-treated groups (Figure 4a, right). These analyses demonstrate that transient selective inhibition of PPARα is sufficient for not only the inhibition of CSC marker expression, but also for the stable inhibition of sphere formation in CSCs.

### 3.5. Genetic Inhibition of PPARα Suppresses Cancer Stemness

**Figure 5:**
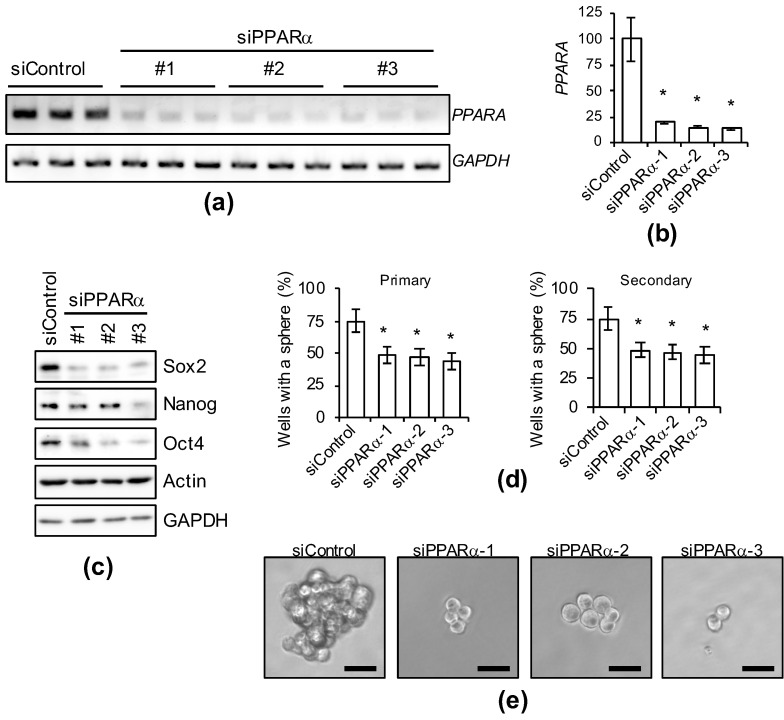
Genetic silencing of PPARα causes the loss of stemness in PANC-1 CSCs. PANC-1 CSCs were transiently transfected with siRNA against PPARα or control siRNA (siControl). After four days, the transfected cells were subjected to each analysis as described below. (**a**–**d**) Cells treated as indicated were subjected to agarose gel electrophoresis (**a**), quantification of PCR analyses by densitometry (**b**), immunoblot analyses (**c**), or serial sphere formation analyses (**d**). The values are presented as the means ± SDs from triplicate samples of a representative experiment. Similar results were obtained from two independent biological replicates. (**e**) Representative photographs of primary spheres are shown. Similar results were obtained from three independent biological replicates. Bar: 50 μm. * *p* < 0.05 vs. control siRNA-transfected cells by Dunnett’s test.

As PPARα activity was confirmed to be essential for the expression of CSC markers and maintenance of sphere-formation ability in the experiment using antagonists, we used several siRNAs against PPARα to examine whether the genetic inhibition of PPARα expression exhibits effects similar to those of PPARα antagonists (Figure 5). PPARα gene-selective knockdown suppressed the expression of several stem cell markers (Figure 5c) and the sphere-formation ability was inhibited for a prolonged period (Figure 5d,e). Therefore, PPARα is necessary for the maintenance of cancer stemness.

### 3.6. Inhibition of TAG Turnover Suppresses Cancer Stemness

**Figure 6:**
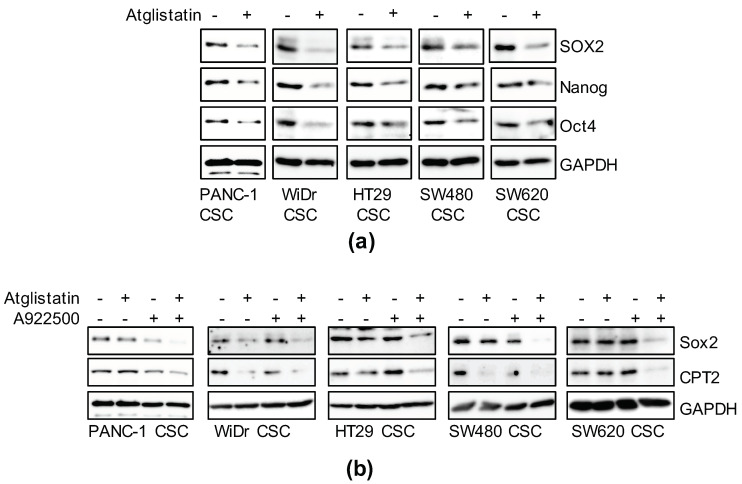
The essential role of triglyceride metabolism in the maintenance of cancer stemness. (**a**) Cells treated with 50 μM Atglistatin for six days were subjected to immunoblot analyses as indicated. (**b**) Cells treated with 10 μM Atglistatin and 5 μM A922500 as described above were subjected to immunoblot analyses for the related proteins. Similar results were obtained from two independent biological replicates.

Thus far, our study suggests that cancer stemness is maintained through activation of the PPARα. Adipose triglyceride lipase (ATGL) is a rate-limiting enzyme of TAG hydrolysis that activates PPARα by producing fatty acids from TAGs [33,34]. Using a recently developed specific ATGL inhibitor, Atglistatin [35], we investigated whether TAG lipolysis is involved in the maintenance of cancer stemness. Atglistatin suppressed the expression of CSC markers, similar to PPARα antagonists, as shown in Figure 3 (Figure 6a). ATGL activation leads to a high rate of lipolysis, resulting in fewer LDs [36]. However, well-developed LDs were maintained more in CSCs than in non-CSCs (Figure 1), suggesting that re-esterification is also increased in CSCs to promote LD accumulation. Thus, we assessed whether cancer stemness can be effectively suppressed by the overall inhibition of TAG turnover by simultaneously treating cells with Atglistatin and A922500—an inhibitor of diacylglycerol O-acyltransferase (DGAT), the enzyme that catalyzes the final step of TAG synthesis [37,38]. When treated with A922500 alone, the inhibitory effects on stemness were low, which may have been due to the presence of residual LD-derived signal, but SOX2 was markedly reduced in combination with low-concentration Atglistatin (Figure 6b). A decrease in CPT2 transcribed by PPARα was simultaneously confirmed (Figure 6b). This suggested that TAG turnover strengthens the maintenance of cancer stemness through PPARα activation.

## 4. Discussion

It was previously reported that LDs are accumulated in CSCs of numerous cancer types, but the role of LDs in maintaining cancer stemness remains unclear [10,18,19,20,21,22]. In this study, we focused on CSCs with essential roles in tumorigenesis and recurrence, and clarified that LDs are more abundant in pancreatic and colorectal CSCs than in isogenic non-CSCs. It is well-known that fatty acids (lipolytic products of TAGs abundantly present in these LDs) are natural lipid ligands of PPARα [24]. Moreover, PPARα promotes the proliferation of certain CSCs and can be used as a marker of malignancy, but the relevance of PPARα to CSC maintenance remains unclear [39,40,41]. We investigated the role of the LD–PPARα axis in the stem cell properties of pancreatic and colorectal CSCs. The LD–PPARα axis was higher in pancreatic and colorectal CSCs than in non-CSCs, and the pharmacological and genetic inhibition of PPARα and its upstream suppressed cancer stemness. To obtain more robust data indicating that the LD–PPARα axis is necessary for the maintenance of cancer stemness, combination experiments of PPARα knockdown/GW6471 or Atglistatin/A922500 with a PPARα agonist (e.g., fenofibrate) may be useful and needed in future consideration. On the other hand, fenofibrate, which is a PPARα agonist (i.e., activates PPARα), is used for hypercholesterolemia, and was reported to inhibit cancer cell proliferation by increasing β-oxidation and inhibiting glycolysis [42,43]. However, there are few reports on its anti-tumor effects in CSCs. As the energy production of CSCs relies on glycolysis, OXPHOS, and increased β-oxidation [1,2,3,4,5], the effects of PPARα activation on the inhibition of CSC properties and proliferation may be markedly low. Indeed, fenofibrate was previously reported to induce cell death in a PPARα-independent manner and to reduce the expression level of the stem-cell markers Oct4/CD133 in glioblastoma but the concentration in this experiment (25–100 μM) largely exceeded the clinically valid concentration (Cmax = 3 μg/mL: up to 10 μM) [44]. On the other hand, no clinical study on a PPARα antagonist has been performed, and there have been few reports on the use of GW6471—one of the PPARα-specific antagonists used in this study—in vivo. Another PPARα antagonist, NXT629, inhibited the enlargement of chronic lymphocytic leukemia and ovarian cancer in a mouse model, and only NXT629 administration immediately after transplantation exhibited anti-tumor effects in a B16F10 melanoma subcutaneous transplantation model [45,46]. Taken together with our findings, the PPARα activity may be essential for the in vivo development of certain types of tumors and the early tumor engraftment reaction after metastasis.

In the present study, we did not identify which downstream factors/targets of PPARα are important for the maintenance of CSC properties. As SCD1 was reported to act as a functional downstream factor of PPARα and is involved in the maintenance of CSCs in hepatocellular carcinoma (HCC), the PPARα–SCD1 axis may be one of the important pathways in the maintenance of CSCs [39]. It was also reported that CPT1A and CPT2—known target genes of PPARα—are involved in the radiation resistance of breast cancer stem cells and overall survival of breast cancer [47]. Further analyses using the inhibitors of downstream targets such as the CPT1A inhibitor etximor are required to elucidate the underlying mechanism of LD–PPARα-mediated maintenance of CSC properties. We roughly eliminated the possibility of PPARβ/δ and PPARγ being involved in cancer stemness using GSK3787/GW9662, and suggested that PPARα is necessary for the maintenance of stemness in pancreatic and colorectal CSCs. However, we have not completely excluded the possibility that PPARβ/δ and PPARγ may be involved in CSC maintenance, which suggests that the knockdown of each gene and analyses with more selective antagonists (e.g., GSK0660, ST247, PT-S264) are necessary in further studies. As previous reports demonstrated that other PPARs play important roles in maintaining the stemness of other CSCs [48,49,50], PPARα, PPARβ/δ, and PPARγ may be important for certain CSC maintenance mechanisms.

Excessive development of LDs and the accumulation of fatty acids are involved in not only obesity and insulin resistance, but also carcinogenesis [13]. It was previously reported that CSCs—a key player in tumorigenesis—accumulate more LDs than non-CSCs [10,18,19,20,21,22], and we confirmed that LDs developed more in colorectal and pancreatic CSCs than in isogenic non-CSCs, as described above. Consistent with previous studies, the number of small–medium-sized LDs was higher in CSCs than in isogenic non-CSCs. However, there were no giant LDs, such as those observed in adipocytes, in CSCs. In addition, simultaneous inhibition of DGAT and ATGL, which catalyze the biosynthesis and lipolysis of TAG, respectively [34,37,38,51], suppressed the CSC properties. Based on these findings, LDs in CSCs may not accumulate excess lipids passively, but instead supply lipids actively through high-rate lipid turnover. To maintain cancer tissue in an easily changing surrounding environment, the energy metabolism of CSCs is highly flexible. For example, OXPHOS and autophagy (including lipophagy) in CSCs are high, making them tolerant to nutritional deficiency and environment-dependent stress [4]. Moreover, CSCs incorporate fatty acids and accumulate LDs in a HIF1α-dependent manner under hypoxic stress, and fatty acid synthesis is continued via acetyl-CoA synthesis by acetyl-CoA synthase 2 [52,53]. The presence of many LDs may increase the metabolic stress-buffering capacity of CSCs. Therefore, to silence CSCs, suppression of an energy production pathway may not be sufficient, and safe suppression of several pathways may be necessary. For example, concomitant inhibition of pathways involved in re-esterification and fatty acid degradation—in addition to metabolic stress by inhibiting many of the previously discovered glycolytic factors, including metformin [54,55,56,57]—may be effective. Furthermore, as obesity increases the plasma free fatty acid level, PPARα may be activated LD-dependently, non-dependently, and persistently. As the increase in PPARα transcription activity induces the expression of lipolytic factors such as ATGL [32], a positive feedback loop of fatty-acid metabolic turnover may be formed, which may function in stem-cell maintenance or production, increasing the risk of carcinogenesis. As such, the inhibition of PPARα may be optimal as a target for CSC inhibition.

## 5. Conclusions

The present study clarified that the number of LDs in pancreatic and colorectal CSCs was higher than that in non-CSCs. PPARα activation, which is considered a downstream factor of LD-derived signaling, was observed in CSCs, and pharmacological or genetic PPARα inhibition suppressed CSC properties in pancreatic and colorectal CSC models. Of note, simultaneous inhibition of TAG synthesis and lipolysis, which produce fatty acids from LDs and activate PPARα, was more effective in reducing cancer stemness. Our study provides important insight into the relationship between CSC control and the LD–PPARα axis.

## Data Availability

All data is contained the article and there is no repository data.
